# Supporting health and well-being among infants born to First Nations parents experiencing incarceration: a partnership-based whole-population administrative data study.

**DOI:** 10.23889/ijpds.v7i3.1948

**Published:** 2022-08-25

**Authors:** Nathan C. Nickel, Wanda Phillips-Beck

**Affiliations:** 1 Manitoba Centre for Health Policy, University of Manitoba; 2 First Nations Health and Social Secretariat of Manitoba

## Objectives

Generations of racist and colonial policies have resulted in First Nations (FN) people being systematically over-represented in Canada’s legal system. FN researchers partnered with data scientists at the University of Manitoba to document the birth outcomes associated with experiences of prenatal incarceration among FN families.

## Approach

This retrospective cohort study linked whole-population administrative data from (i) Manitoba’s legal system to identify infants born to people incarcerated while pregnant, (ii) the First Nations research file to identify FN families, (iii) hospital records for birth outcomes, (iii) health and social services data for measured confounders. All Manitoba residents with a live birth (Jan 2004 - Dec 2017), and their infants, were eligible. Generalized linear models tested for differences in birth outcomes associated with experiencing incarceration while pregnant. Propensity score weights adjusted for measured confounders. Effect modification analyses tested whether associations differed between FN and all other Manitobans (AOM).

## Results

FN people were more likely to experience incarceration while pregnant (n=1449) than AOM (n=278). Before propensity score adjustment, incarcerated pregnant people differed on important sociodemographic confounding characteristics from pregnant people who were not incarcerated – e.g., lower socioeconomic status, higher prevalence of pre-existing mental disorders, higher prevalence of having a previous child taken into care of family services, more likely to live in an urban setting. After propensity score adjustment, confounding characteristics were balanced between exposure groups. After adjustment, infants born to people incarcerated while pregnant were more likely to be low birth weight at term (aRR 1.76; 95% CI 1.41-2.18), be born preterm (aRR 1.44; 1.33-1.56), be small for gestational age (aRR 1.40; 1.28-1.54). Associations did not differ between FN and AOM families.

## Conclusion

Incarceration of pregnant people compromises their infant’s birth outcomes and perpetuates intergenerational systems of oppression that exacerbate health inequities. To improve the health and well-being of FN people, we must implement Calls to Action outlined by the Truth and Reconciliation Commission to redress these harms experienced by FN people.

